# Deacetyl tenuazonic acid *p*-toluene­sulfonyl­hydrazone

**DOI:** 10.1107/S1600536809048958

**Published:** 2009-11-21

**Authors:** David Siegel, Franziska Blaske, Matthias Koch, Franziska Emmerling, Irene Nehls

**Affiliations:** a BAM Federal Institute for Materials Research and Testing, Abteilung Analytische Chemie; Referenzmaterialien, Richard-Willstätter-Strasse 11, D-12489 Berlin-Adlershof, Germany

## Abstract

The title compound {systematic name: 4-methyl-*N*′-[(3*E*)-2-(1-methyl­prop­yl)-5-oxopyrrolidin-3-yl­idene]benzene­sulfono­hydrazide}, C_15_H_21_N_3_O_3_S, is the condensation product of deacetyl tenuazonic acid (DTA) and *p*-toluene­sulfonohydrazide. The crystal structure consists of chains along [100] linked by N—H⋯O hydrogen bonds.

## Related literature

For the occurrence of tenuazonic acid (TA) in various food matrices, see: Weidenbörner (2001 ▶). For potential uses of the title compound in food analysis, see: Siegel, Rasenko *et al.* (2009 ▶). For the crystal structure of DTA, see: Siegel, Koch *et al.* (2009 ▶) and for its synthesis, see: Lebrun *et al.* (1988 ▶); Stickings (1959 ▶). For the structure of *p*-toluene­sulfonyl­hydrazine, see: Roy & Nangia (2007 ▶). For the structures of other *p*-toluene­sulfonyhydrazones, see, for example: Glidewell *et al.* (2004 ▶); Ng (1997 ▶); Yan *et al.* (2008 ▶).
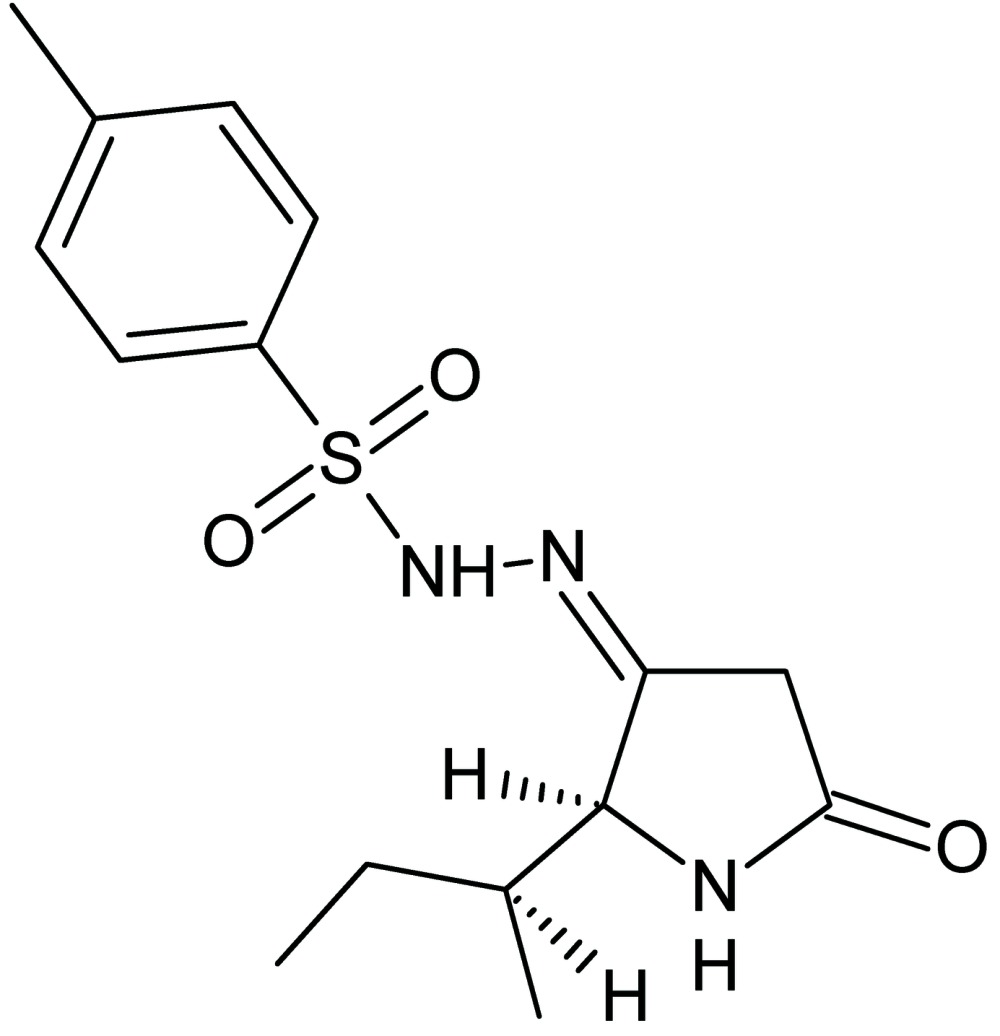


## Experimental

### 

#### Crystal data

C_15_H_21_N_3_O_3_S
*M* *_r_* = 323.41
Orthorhombic, 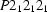

*a* = 5.1286 (16) Å
*b* = 8.285 (3) Å
*c* = 38.430 (12) Å
*V* = 1633.0 (9) Å^3^
*Z* = 4
Mo *K*α radiation
μ = 0.21 mm^−1^
*T* = 294 K
0.14 × 0.12 × 0.02 mm

#### Data collection

Bruker APEX CCD area-detector diffractometer
Absorption correction: ψ scan (*SHELXTL*; Bruker, 2001 ▶) *T*_min_ = 0.970, *T*_max_ = 0.995
13112 measured reflections
4755 independent reflections
2233 reflections with *I* > 2σ(*I*)
*R*_int_ = 0.102

#### Refinement

*R*[*F* ^2^ > 2σ(*F* ^2^)] = 0.045
*wR*(*F* ^2^) = 0.097
*S* = 0.77
4755 reflections
196 parameters
H-atom parameters constrained
Δρ_max_ = 0.21 e Å^−3^
Δρ_min_ = −0.29 e Å^−3^
Absolute structure: Flack (1983 ▶), 1905 Friedel pairs
Flack parameter: −0.11 (10)

### 

Data collection: *SMART* (Bruker, 2001 ▶); cell refinement: *SAINT* (Bruker, 2001 ▶); data reduction: *SAINT*; program(s) used to solve structure: *SHELXS97* (Sheldrick, 2008 ▶); program(s) used to refine structure: *SHELXL97* (Sheldrick, 2008 ▶); molecular graphics: *SHELXTL* (Sheldrick, 2008 ▶) and *ORTEPIII* (Burnett & Johnson, 1996 ▶); software used to prepare material for publication: *SHELXTL*.

## Supplementary Material

Crystal structure: contains datablocks I, global. DOI: 10.1107/S1600536809048958/sj2674sup1.cif

Structure factors: contains datablocks I. DOI: 10.1107/S1600536809048958/sj2674Isup2.hkl

Additional supplementary materials:  crystallographic information; 3D view; checkCIF report

## Figures and Tables

**Table 1:** Hydrogen-bond geometry (Å, °)

*D*—H⋯*A*	*D*—H	H⋯*A*	*D*⋯*A*	*D*—H⋯*A*
N3—H3⋯O1^i^	0.86	2.27	3.104 (3)	162
N1—H1⋯O3^ii^	1.03	2.10	3.063 (3)	156

Symmetry codes: (i) 
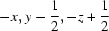
; (ii) 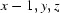.

**Fig. 1.**
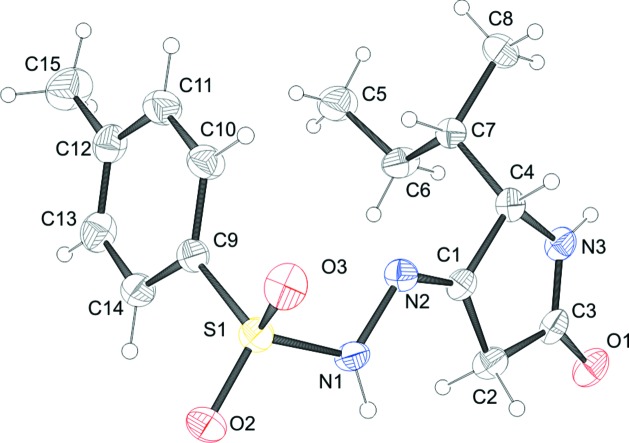
The structure of the title compound with labelling and displacement ellipsoids drawn at the 50% probability level.

**Fig. 2.**
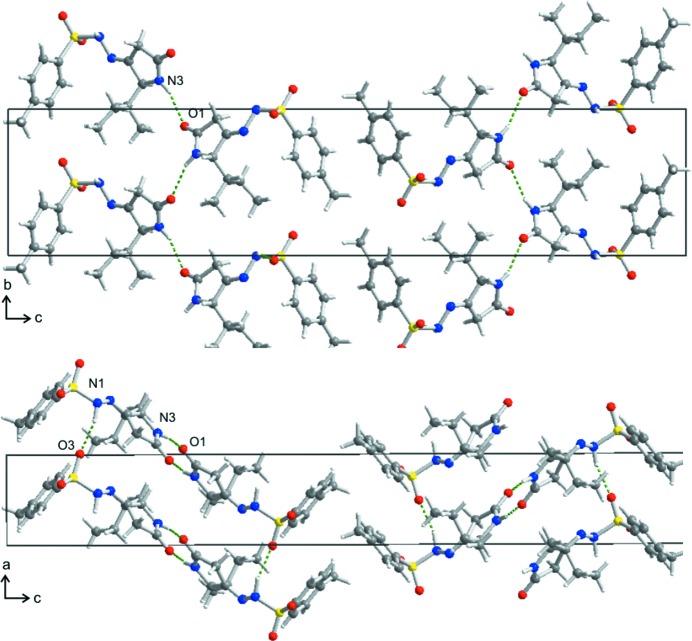
View of the unit cell of the title compound along [100] (upper picture) and [010] (lower picture) showing the hydrogen bond system drawn as dashed lines.

## supplementary crystallographic information

### Comment

Deacetyl tenuazonic acid (DTA) is formed upon boiling the *Alternaria.* mycotoxin tenuazonic acid (TA) in 0.1 *M* HCl (Stickings, 1959). As TA is frequently encountered in various food matrices (Weidenbörner, 2001), traces of DTA are expected to occur in these matrices as well. We have recently reported a derivatization procedure for TA quantification in food, which is based on hydrazone formation with 2,4-dinitrophenylhydrazine (Siegel, Rasenko *et al.*, 2009) and are currently evaluating a similar procedure for DTA quantification using *p*-toluenesulfonyl hydrazide. The title compound is the product of the latter derivatization reaction. The stucture of the title compound, (I), is shown below. Each molecule (Fig.1) is connected to three adjacent molecules *via* N—H···O hydrogen bonds. As a result isolated ribbons are formed along the *a* axis, as depicted in Fig. 2.

### Experimental

DTA was supplied by the workgroup of Professor *R*. Faust (University of Kassel, Germany) by synthesis according to a literature procedure (Lebrun *et al.*, 1988). Its identity was confirmed by *x*-ray crystallography (Siegel, Koch*et al.*, 2009). The title compound was synthesized by dissolving 20 mg (1 eq., 0.13 mmol) of DTA and a five fold molar excess of *p*-toluenesulfonyl hydrazide (5 eq., 0.65 mmol, 121 mg) in 50 ml 2 *M* HCl. After 30 minutes of shaking the precipitate was collected, washed with water, dissolved in ethyl acetate and dried with sodium sulfate. After evaporation of the solvent, a yellow powder was obtained, which was recrystallized from ethanol twice. For single-crystal *x*-ray crystallography, orange crystals of the title compound were grown by solvent evaporation (methanol:water 50:50 v:v) at ambient temperature over a period of three weeks.

### Refinement

All non-hydrogen atoms were refined anisotropically. The hydrogen atoms were located in difference maps but positioned with idealized geometry and refined using the riding model,with C—H = 0.98–1 Å or N—H = 0.9 Å and *U*_iso_(H) = 1.2 *U*_eq_ (C, N) or 1.5 *U*_eq_(C_methyl_).

### Crystal data

**Table:**

C_15_H_21_N_3_O_3_S	*F*(000) = 688
*M**_r_* = 323.41	*D*_x_ = 1.315 Mg m^−^^3^
Orthorhombic, *P*2_1_2_1_2_1_	Mo *K*α radiation, λ = 0.71073 Å
Hall symbol: P 2ac 2ab	Cell parameters from 122 reflections
*a* = 5.1286 (16) Å	θ = 4–23°
*b* = 8.285 (3) Å	µ = 0.21 mm^−^^1^
*c* = 38.430 (12) Å	*T* = 294 K
*V* = 1633.0 (9) Å^3^	Needle, colourless
*Z* = 4	0.14 × 0.12 × 0.02 mm

### Data collection

**Table:**

Bruker APEX CCD area-detector diffractometer	4755 independent reflections
Radiation source: fine-focus sealed tube	2233 reflections with *I* > 2σ(*I*)
graphite	*R*_int_ = 0.102
ω/2θ scans	θ_max_ = 31.1°, θ_min_ = 2.1°
Absorption correction: ψ scan (*SHELXTL*; Bruker, 2001)	*h* = −7→7
*T*_min_ = 0.970, *T*_max_ = 0.995	*k* = −12→12
13112 measured reflections	*l* = −55→55

### Refinement

**Table:**

Refinement on *F*^2^	Secondary atom site location: difference Fourier map
Least-squares matrix: full	Hydrogen site location: inferred from neighbouring sites
*R*[*F*^2^ > 2σ(*F*^2^)] = 0.045	H-atom parameters constrained
*wR*(*F*^2^) = 0.097	*w* = 1/[σ^2^(*F*_o_^2^) + (0.0333*P*)^2^] where *P* = (*F*_o_^2^ + 2*F*_c_^2^)/3
*S* = 0.77	(Δ/σ)_max_ = 0.008
4755 reflections	Δρ_max_ = 0.21 e Å^−^^3^
196 parameters	Δρ_min_ = −0.28 e Å^−^^3^
0 restraints	Absolute structure: Flack (1983), 1905 Friedel pairs
Primary atom site location: structure-invariant direct methods	Flack parameter: −0.11 (10)

### Special details

**Table:**

Geometry. All e.s.d.'s (except the e.s.d. in the dihedral angle between two l.s. planes) are estimated using the full covariance matrix. The cell e.s.d.'s are taken into account individually in the estimation of e.s.d.'s in distances, angles and torsion angles; correlations between e.s.d.'s in cell parameters are only used when they are defined by crystal symmetry. An approximate (isotropic) treatment of cell e.s.d.'s is used for estimating e.s.d.'s involving l.s. planes.
Refinement. Refinement of *F*^2^ against ALL reflections. The weighted *R*-factor *wR* and goodness of fit *S* are based on *F*^2^, conventional *R*-factors *R* are based on *F*, with *F* set to zero for negative *F*^2^. The threshold expression of *F*^2^ > σ(*F*^2^) is used only for calculating *R*-factors(gt) *etc*. and is not relevant to the choice of reflections for refinement. *R*-factors based on *F*^2^ are statistically about twice as large as those based on *F*, and *R*- factors based on ALL data will be even larger.

### Fractional atomic coordinates and isotropic or equivalent isotropic displacement parameters (Å^2^)

**Table:**

	*x*	*y*	*z*	*U*_iso_*/*U*_eq_	
S1	0.74721 (13)	0.49291 (7)	0.097917 (14)	0.04011 (15)	
O1	−0.0629 (4)	0.3828 (2)	0.23977 (4)	0.0606 (5)	
O2	0.6752 (4)	0.63657 (18)	0.08020 (4)	0.0527 (5)	
O3	1.0099 (3)	0.4666 (2)	0.10814 (4)	0.0519 (5)	
N1	0.5727 (4)	0.4954 (2)	0.13411 (4)	0.0390 (4)	
H1	0.3769	0.5132	0.1302	0.047*	
N2	0.6024 (4)	0.3487 (2)	0.15275 (5)	0.0383 (5)	
N3	0.2395 (4)	0.1958 (2)	0.22262 (5)	0.0448 (5)	
H3	0.1936	0.1221	0.2371	0.054*	
C1	0.4445 (5)	0.3286 (3)	0.17784 (6)	0.0347 (5)	
C2	0.2331 (6)	0.4344 (3)	0.19185 (6)	0.0462 (6)	
H2A	0.3038	0.5354	0.2005	0.055*	
H2B	0.1039	0.4574	0.1741	0.055*	
C3	0.1172 (5)	0.3376 (3)	0.22082 (6)	0.0445 (6)	
C4	0.4556 (5)	0.1730 (3)	0.19850 (6)	0.0364 (6)	
H4	0.6190	0.1700	0.2117	0.044*	
C5	0.4380 (4)	0.0220 (3)	0.17583 (5)	0.0366 (5)	
H5	0.5686	0.0339	0.1574	0.044*	
C6	0.1756 (4)	0.0098 (3)	0.15812 (6)	0.0542 (7)	
H6A	0.1396	0.1114	0.1465	0.065*	
H6B	0.0428	−0.0054	0.1758	0.065*	
C7	0.1531 (6)	−0.1253 (4)	0.13175 (7)	0.0741 (10)	
H7A	0.2885	−0.1145	0.1147	0.111*	
H7B	−0.0139	−0.1197	0.1205	0.111*	
H7C	0.1703	−0.2274	0.1433	0.111*	
C8	0.5103 (4)	−0.1307 (2)	0.19639 (5)	0.0529 (8)	
H8A	0.6778	−0.1162	0.2071	0.079*	
H8B	0.5166	−0.2214	0.1809	0.079*	
H8C	0.3816	−0.1498	0.2141	0.079*	
C9	0.6413 (3)	0.32472 (18)	0.07366 (4)	0.0374 (6)	
C10	0.7575 (3)	0.17800 (18)	0.07827 (4)	0.0494 (6)	
H10	0.8985	0.1674	0.0933	0.059*	
C11	0.6641 (6)	0.0458 (3)	0.06047 (7)	0.0565 (8)	
H11	0.7440	−0.0539	0.0637	0.068*	
C12	0.4556 (6)	0.0576 (3)	0.03813 (7)	0.0529 (7)	
C13	0.3442 (6)	0.2071 (4)	0.03385 (7)	0.0593 (8)	
H13	0.2038	0.2183	0.0187	0.071*	
C14	0.4359 (5)	0.3420 (3)	0.05156 (6)	0.0488 (7)	
H14	0.3583	0.4424	0.0483	0.059*	
C15	0.3523 (7)	−0.0880 (4)	0.01904 (8)	0.0868 (11)	
H15A	0.4174	−0.0880	−0.0044	0.130*	
H15B	0.1652	−0.0843	0.0186	0.130*	
H15C	0.4083	−0.1844	0.0307	0.130*	

### Atomic displacement parameters (Å^2^)

**Table:**

	*U*^11^	*U*^22^	*U*^33^	*U*^12^	*U*^13^	*U*^23^
S1	0.0445 (3)	0.0340 (3)	0.0418 (3)	−0.0070 (4)	0.0058 (3)	0.0019 (3)
O1	0.0621 (12)	0.0658 (13)	0.0540 (11)	−0.0020 (10)	0.0229 (11)	−0.0140 (9)
O2	0.0702 (14)	0.0343 (10)	0.0538 (10)	−0.0042 (9)	0.0046 (10)	0.0118 (8)
O3	0.0363 (9)	0.0603 (12)	0.0590 (10)	−0.0088 (9)	0.0040 (8)	−0.0056 (9)
N1	0.0412 (10)	0.0320 (10)	0.0438 (10)	−0.0030 (11)	0.0070 (9)	0.0008 (10)
N2	0.0399 (11)	0.0344 (11)	0.0405 (11)	−0.0056 (9)	0.0043 (11)	0.0034 (9)
N3	0.0547 (13)	0.0410 (12)	0.0387 (11)	−0.0104 (13)	0.0126 (12)	0.0005 (9)
C1	0.0347 (13)	0.0331 (13)	0.0364 (13)	−0.0067 (11)	−0.0005 (12)	−0.0063 (11)
C2	0.0526 (16)	0.0364 (13)	0.0497 (14)	−0.0069 (14)	0.0110 (14)	−0.0034 (10)
C3	0.0502 (16)	0.0458 (17)	0.0375 (14)	−0.0101 (14)	0.0051 (13)	−0.0131 (12)
C4	0.0351 (14)	0.0401 (14)	0.0339 (13)	−0.0102 (12)	0.0025 (11)	0.0021 (11)
C5	0.0367 (13)	0.0353 (14)	0.0378 (12)	0.0002 (12)	0.0025 (10)	0.0004 (11)
C6	0.0496 (16)	0.0443 (15)	0.0685 (17)	0.0041 (14)	−0.0143 (13)	−0.0161 (15)
C7	0.088 (3)	0.0531 (19)	0.082 (2)	0.0056 (17)	−0.0259 (19)	−0.0128 (16)
C8	0.061 (2)	0.0438 (17)	0.0543 (17)	−0.0015 (14)	0.0013 (14)	0.0120 (13)
C9	0.0390 (15)	0.0374 (14)	0.0357 (14)	−0.0051 (12)	0.0044 (12)	0.0051 (11)
C10	0.0548 (16)	0.0423 (14)	0.0511 (15)	0.0011 (17)	−0.0117 (15)	0.0008 (12)
C11	0.071 (2)	0.0400 (17)	0.0582 (17)	0.0025 (13)	−0.0077 (15)	0.0011 (12)
C12	0.0618 (19)	0.0510 (18)	0.0460 (16)	−0.0095 (15)	−0.0003 (15)	−0.0076 (13)
C13	0.0557 (19)	0.071 (2)	0.0515 (17)	0.0002 (16)	−0.0091 (15)	−0.0086 (15)
C14	0.0549 (17)	0.0453 (16)	0.0462 (15)	0.0059 (14)	−0.0019 (14)	−0.0006 (12)
C15	0.104 (3)	0.068 (2)	0.089 (2)	−0.014 (2)	−0.015 (2)	−0.0270 (18)

### Geometric parameters (Å, °)

**Table:**

S1—O3	1.4200 (17)	C6—H6A	0.9700
S1—O2	1.4201 (16)	C6—H6B	0.9700
S1—N1	1.6542 (18)	C7—H7A	0.9600
S1—C9	1.7624 (18)	C7—H7B	0.9600
O1—C3	1.235 (3)	C7—H7C	0.9600
N1—N2	1.419 (2)	C8—H8A	0.9600
N1—H1	1.0263	C8—H8B	0.9600
N2—C1	1.270 (3)	C8—H8C	0.9600
N3—C3	1.333 (3)	C9—C10	1.3653
N3—C4	1.457 (3)	C9—C14	1.361 (3)
N3—H3	0.8600	C10—C11	1.377 (3)
C1—C2	1.494 (3)	C10—H10	0.9300
C1—C4	1.515 (3)	C11—C12	1.375 (4)
C2—C3	1.495 (3)	C11—H11	0.9300
C2—H2A	0.9700	C12—C13	1.374 (4)
C2—H2B	0.9700	C12—C15	1.508 (4)
C4—C5	1.527 (3)	C13—C14	1.391 (3)
C4—H4	0.9800	C13—H13	0.9300
C5—C6	1.511 (3)	C14—H14	0.9300
C5—C8	1.537 (3)	C15—H15A	0.9600
C5—H5	0.9800	C15—H15B	0.9600
C6—C7	1.515 (3)	C15—H15C	0.9600
			
O3—S1—O2	120.52 (11)	C5—C6—H6B	108.6
O3—S1—N1	106.41 (10)	C7—C6—H6B	108.6
O2—S1—N1	104.60 (10)	H6A—C6—H6B	107.6
O3—S1—C9	108.51 (10)	C6—C7—H7A	109.5
O2—S1—C9	109.21 (9)	C6—C7—H7B	109.5
N1—S1—C9	106.72 (9)	H7A—C7—H7B	109.5
N2—N1—S1	110.85 (14)	C6—C7—H7C	109.5
N2—N1—H1	107.6	H7A—C7—H7C	109.5
S1—N1—H1	114.1	H7B—C7—H7C	109.5
C1—N2—N1	115.28 (18)	C5—C8—H8A	109.5
C3—N3—C4	116.02 (19)	C5—C8—H8B	109.5
C3—N3—H3	122.0	H8A—C8—H8B	109.5
C4—N3—H3	122.0	C5—C8—H8C	109.5
N2—C1—C2	131.2 (2)	H8A—C8—H8C	109.5
N2—C1—C4	119.1 (2)	H8B—C8—H8C	109.5
C2—C1—C4	109.71 (19)	C10—C9—C14	120.84 (13)
C3—C2—C1	104.0 (2)	C10—C9—S1	120.06 (6)
C3—C2—H2A	111.0	C14—C9—S1	119.04 (15)
C1—C2—H2A	111.0	C9—C10—C11	119.48 (14)
C3—C2—H2B	111.0	C9—C10—H10	120.3
C1—C2—H2B	111.0	C11—C10—H10	120.3
H2A—C2—H2B	109.0	C12—C11—C10	121.6 (2)
O1—C3—N3	126.1 (2)	C12—C11—H11	119.2
O1—C3—C2	125.0 (2)	C10—C11—H11	119.2
N3—C3—C2	108.9 (2)	C11—C12—C13	117.5 (2)
N3—C4—C1	101.24 (19)	C11—C12—C15	121.4 (3)
N3—C4—C5	115.09 (18)	C13—C12—C15	121.1 (3)
C1—C4—C5	113.32 (18)	C12—C13—C14	121.7 (3)
N3—C4—H4	109.0	C12—C13—H13	119.2
C1—C4—H4	109.0	C14—C13—H13	119.2
C5—C4—H4	109.0	C9—C14—C13	118.8 (2)
C6—C5—C4	111.3 (2)	C9—C14—H14	120.6
C6—C5—C8	113.0 (2)	C13—C14—H14	120.6
C4—C5—C8	111.52 (17)	C12—C15—H15A	109.5
C6—C5—H5	106.8	C12—C15—H15B	109.5
C4—C5—H5	106.8	H15A—C15—H15B	109.5
C8—C5—H5	106.8	C12—C15—H15C	109.5
C5—C6—C7	114.8 (2)	H15A—C15—H15C	109.5
C5—C6—H6A	108.6	H15B—C15—H15C	109.5
C7—C6—H6A	108.6		
			
O3—S1—N1—N2	−58.11 (17)	N3—C4—C5—C8	−76.8 (2)
O2—S1—N1—N2	173.28 (14)	C1—C4—C5—C8	167.36 (18)
C9—S1—N1—N2	57.61 (15)	C4—C5—C6—C7	172.9 (2)
S1—N1—N2—C1	−169.08 (16)	C8—C5—C6—C7	−60.6 (3)
N1—N2—C1—C2	−1.3 (4)	O3—S1—C9—C10	24.73 (9)
N1—N2—C1—C4	177.98 (18)	O2—S1—C9—C10	157.88 (8)
N2—C1—C2—C3	178.5 (2)	N1—S1—C9—C10	−89.58 (8)
C4—C1—C2—C3	−0.8 (2)	O3—S1—C9—C14	−158.08 (16)
C4—N3—C3—O1	−177.0 (2)	O2—S1—C9—C14	−24.93 (18)
C4—N3—C3—C2	3.1 (3)	N1—S1—C9—C14	87.61 (17)
C1—C2—C3—O1	178.9 (2)	C14—C9—C10—C11	−0.46 (17)
C1—C2—C3—N3	−1.3 (3)	S1—C9—C10—C11	176.68 (18)
C3—N3—C4—C1	−3.4 (2)	C9—C10—C11—C12	−0.1 (3)
C3—N3—C4—C5	−126.0 (2)	C10—C11—C12—C13	0.6 (4)
N2—C1—C4—N3	−177.1 (2)	C10—C11—C12—C15	−179.3 (2)
C2—C1—C4—N3	2.3 (2)	C11—C12—C13—C14	−0.5 (4)
N2—C1—C4—C5	−53.3 (3)	C15—C12—C13—C14	179.4 (3)
C2—C1—C4—C5	126.1 (2)	C10—C9—C14—C13	0.5 (3)
N3—C4—C5—C6	50.5 (3)	S1—C9—C14—C13	−176.63 (18)
C1—C4—C5—C6	−65.4 (3)	C12—C13—C14—C9	0.0 (4)

### Hydrogen-bond geometry (Å, °)

**Table:**

*D*—H···*A*	*D*—H	H···*A*	*D*···*A*	*D*—H···*A*
N3—H3···O1^i^	0.86	2.27	3.104 (3)	162
N1—H1···O3^ii^	1.03	2.10	3.063 (3)	156

Symmetry codes: (i) −*x*, *y*−1/2, −*z*+1/2; (ii) *x*−1, *y*, *z*.
